# The molecular mechanisms of *Monascus purpureus* M9 responses to blue light based on the transcriptome analysis

**DOI:** 10.1038/s41598-017-05990-x

**Published:** 2017-07-17

**Authors:** Di Chen, Mianhua Chen, Shufen Wu, Zhenjing Li, Hua Yang, Changlu Wang

**Affiliations:** 1 Key Laboratory of Food Nutrition and Safety (Tianjin University of Science and Technology), Ministry of Education, College of Food Engineering and Biotechnology, Tianjin University of Science and Technology, No. 29, 13th Avenue, TEDA, Tianjin, 300457 P. R. China; 2 College of Biological Engineering, Henan University of Technology, No.100, Lianhua Street, High-tech Industrial Development Area, Zhengzhou, 450001 P. R. China

## Abstract

Light is an important environmental factor that regulates various physiological processes of fungi. To thoroughly study the responses of *Monascus* to blue light, transcriptome sequencing was performed on mRNAs isolated from samples of *Monascus purpureus* M9 cultured under three conditions: darkness (D); exposure to blue light for 15 min/d (B15); and exposure to blue light for 60 min/d over 8 days (B60). The number of differentially expressed genes between the three pairs of samples—B15 vs D, B60 vs B15, and B60 vs D—was 1167, 1172, and 220, respectively. KEGG analysis showed the genes involved in primary metabolism including carbon and nitrogen metabolism were downregulated by B15 light treatment, whereas B15 upregulated expression of genes involved with aromatic amino acid metabolism, which associated with development, and branched chain amino acid metabolism, and fatty acid degradation, which can produce the biosynthetic precursors of pigments. When exposed to B60 conditions, genes with roles in carbohydrate metabolism and protein synthesis were upregulated as part of a stress response to blue light. Based on this study, we propose a predicted light-stimulated signal transduction pathway in *Monascus*. Our work is the first comprehensive investigation concerning the mechanism of *Monascus* responses to blue light.

## Introduction

The genus *Monascus* belongs to the class Ascomycetes, the order Eurotiales, and the family Monascaceae^1^. Red mold rice, the famous fermented product of *Monascus*, has been used as a folk medicine, food colorant, and fermentation starter for more than one thousand years in China^2^. *Monascus* can synthesize many beneficial secondary metabolites, such as *Monascus* pigments (natural food colorants), monacolin K (an anti-hypercholesterolemic agent), γ-GABA (a kind of hypotensive agent), and dimerumic acid (a natural antioxidant)^3–5^. *Monascus* pigments are not only as natural food colorants widely utilized in food industry, but possess a range of biological activities, such as anticancer, antimicrobial, and anti-obesity properties^6–8^.

Light, an important environmental signal, influences various physiological processes of fungi^9^. The effects of light in *Neurospora crassa* include development of asexual spores and sexual structures, biosynthesis of photoprotective pigments in mycelia, and entrainment of the circadian clock^10^. Light also regulates development and secondary metabolism of various *Aspergillus* species, such as *A. nidulans*, *A. fumigatus*, and *A. flavus* ^11, 12^. *Monascus*, whose species are closely related to those of *Aspergillus*, also respond to light. Our previous study found that blue light significantly affected spore germination, mycelia growth, and pigment biosynthesis^13, 14^. However, even after extensive research^15–17^, little is known about the mechanisms of light-induced regulation in *Monascus*.

A complete light signaling pathway has been thorough researched in *N. crassa*, a eukaryotic model system for studying blue-light responses. *Neurospora* perceives blue light through the photoreceptor and GATA zinc finger transcription factor encoded by *white collar-1* (*wc-1*)^18^. WC-1 interacts with the zinc finger protein WC-2 through its Per-Arnt-Sim (PAS) domain to form a heterodimeric transcription factor, the White Collar Complex (WCC)^19^. Upon exposure to light, the WCC binds to light-responsive elements (LREs) in the promoters to activate light-responsive genes transcription^20^. Such genes are involved in various physiological processes constituting a regulatory network in *N. crassa* ^10^. Few papers have reported light regulatory mechanisms in *Monascus*.

With the rapid development and reduction in cost of next generation sequencing, RNA-seq has been widely used to characterize differentially expressed genes under various conditions^21, 22^. Wu *et al*. measured changes in mRNA levels of light-regulated genes while studying the responses of *N. crassa* to light^23^. Several candidate genes and signaling pathways associated with light-induced brown film formation in *Lentinula edodes* have also been analyzed via RNA-seq^24^. In this study, three groups of mRNAs were isolated from the mycelia of *M. purpureus* M9 samples that were cultivated under one of three conditions: grown in the dark (D); exposed to blue light for 15 min/d (B15); or exposed to blue light for 60 min/d (B60). All samples were grown in their respective conditions for eight days, after which their corresponding transcripts were identified by high throughput sequencing and analyzed using digital gene expression profiles. More than one thousand differentially expressed genes (DEGs) were found to respond to blue light and to exert widespread effects on the metabolism of M9. This work was the first investigation concerning the genome-wide response of *Monascus* to blue light. Our results will facilitate an understanding of its light-stimulated regulatory mechanisms.

## Results

### Sequencing and de novo assembly

To better survey the molecular mechanism of blue light-induced transcription in *Monascus*, two biological replicates of each condition were obtained, with the B15 and B60 treatments labeled the “early” and “late” light response, respectively. Capped and polyadenylated mRNA was purified from mycelia, after which cDNA was produced and sequenced; a total of 24–33 million reads were obtained from the six samples. All error rates were below 0.01%, while GC content accounted for about 52% of sequenced bases, indicating highly accurate sequencing data (Supplementary Data S1). Homology analysis showed that M9 is closely related to *M. purpureus* YY-1, so we chose to use the published genome of YY-1 as a reference (Supplementary Fig. S1). The total mapped reads of all six samples possessed greater than 70% sequence identity with respect to the reference genome, again corroborating the validity of our data (Supplementary Data S2)^25^. The strong correlation (R^2^ > 0.97) between biological replicates suggested excellent reproducibility of results for cultures grown under the three different conditions (Supplementary Fig. S2).

### Analysis of differentially expressed genes

**Figure 1:**
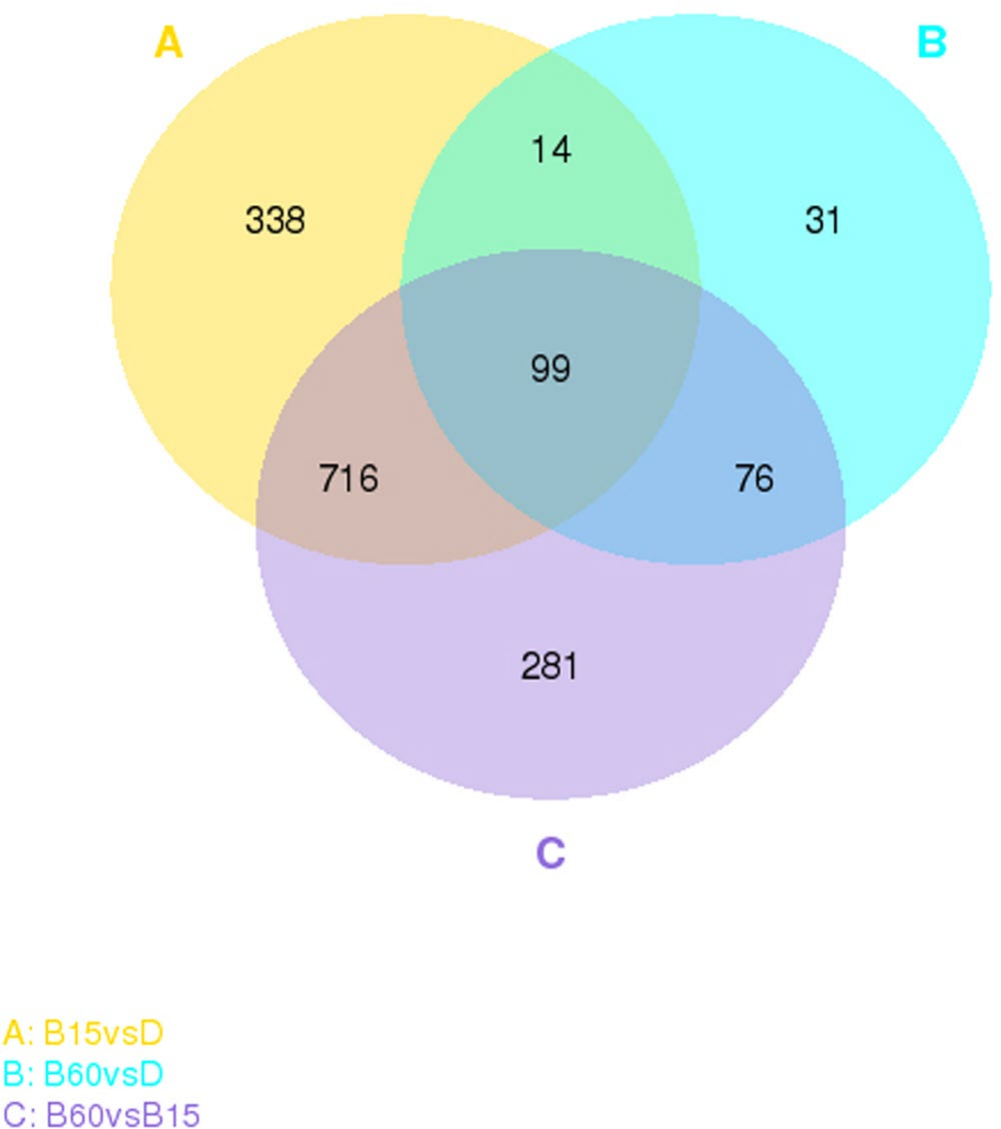
Venn diagram of sequence samples of *M. purpureus* M9. The diagram displays the number of unique and shared differentially expressed genes (DEGs) in the three sets of transcripts obtained from mycelia that were grown in the dark (D); exposed to blue light for 15 min/d (B15); and exposed to blue light for 60 min/d (B60). The number in one color represents the unique DEGs of one comparative group. The number in two overlapping colors represents the shared DEGs of two comparative groups. The number in three overlapping colors represents the shared DEGs of three comparative groups.

**Figure 2:**
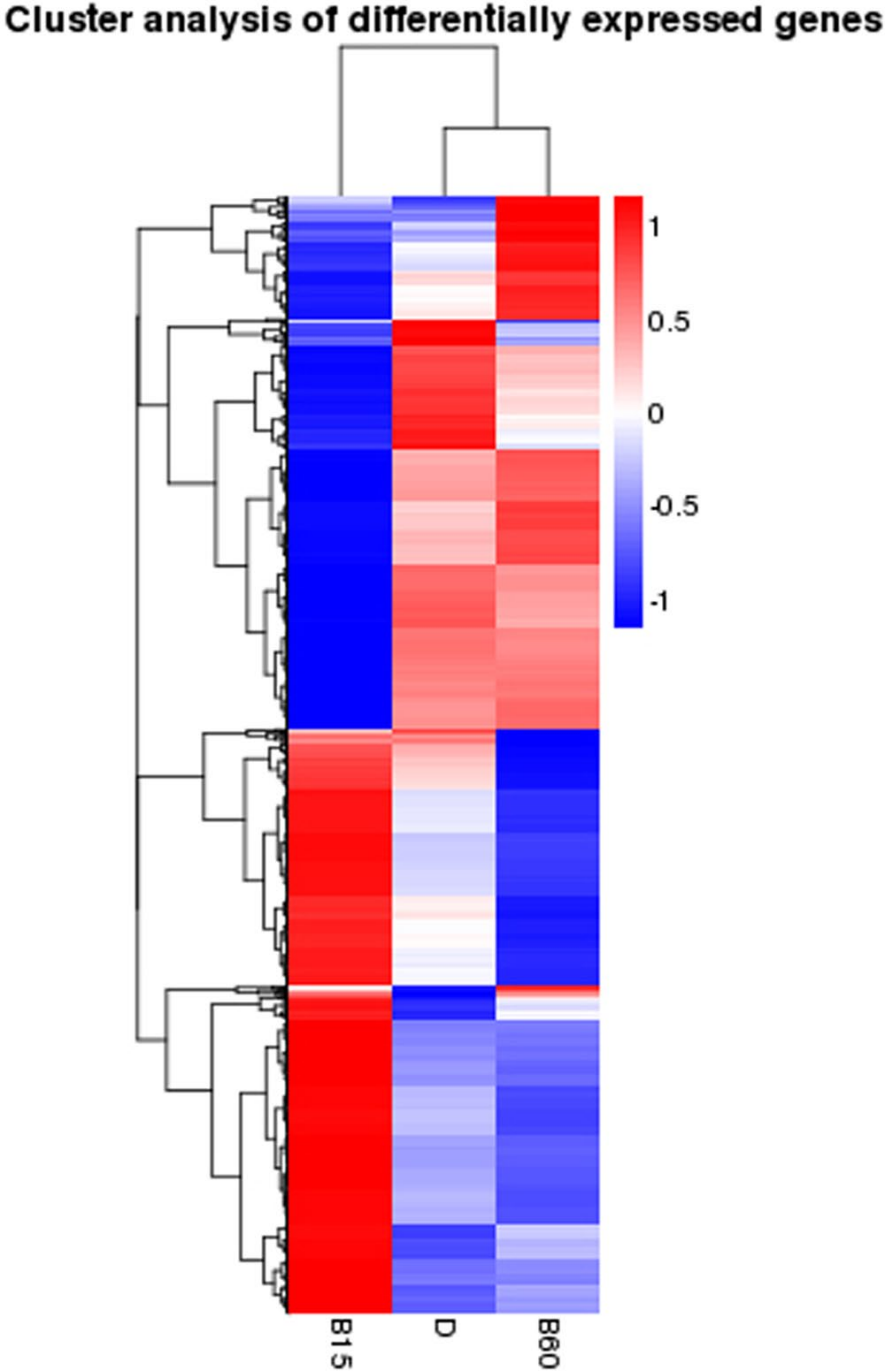
Cluster analysis of differentially expressed genes of *M. purpureus* M9. Unsupervised hierarchical clustering of DEGs representing transcripts obtained from mycelia that were: grown in the dark (D); exposed to blue light for 15 min/d (B15); and exposed to blue light for 60 min/d (B60). The number of reads corresponding to each gene was divided by log_10_ (FPKM + 1). Red: upregulated; blue: downregulated.

About 93% of genes were expressed with fragments per kilo base per million reads (FPKM) > 1 at one or more of the time points analyzed; these were considered “expressed genes” (Supplementary Data S3). A Venn diagram illustrates that 1167, 1172, and 220 DEGs were found amongst the three sample groups: “B15 vs D”, “B60 vs B15” and “B60 vs D”, respectively (Fig. 1). The number of unique DEGs belonging to B15 vs D (338) and B60 vs B15 (281) was much greater than the number belonging to B60 vs D (31), which indicated that the B15 light treatment induced expression of more genes than did B60 when compared to D (Fig. 1). Similar results were observed in a cluster analysis of DEGs: in terms of gene expression, there were relatively large differences between B15 and D, whereas expression in B60 was almost the same as in D. B15 upregulated/downregulated the expression of genes that were downregulated/upregulated under B60 (Fig. 2). It may be that M9 took stress response to short-term blue light stimulus (B15 vs D), so a large number of genes expression were regulated, and then when exposure time was extended to B60, it recovered genes expressions to the D level for adapting blue light (B60 vs B15).

### GO functional classification and KEGG pathway enrichment of differentially expressed genes

**Figure 3:**
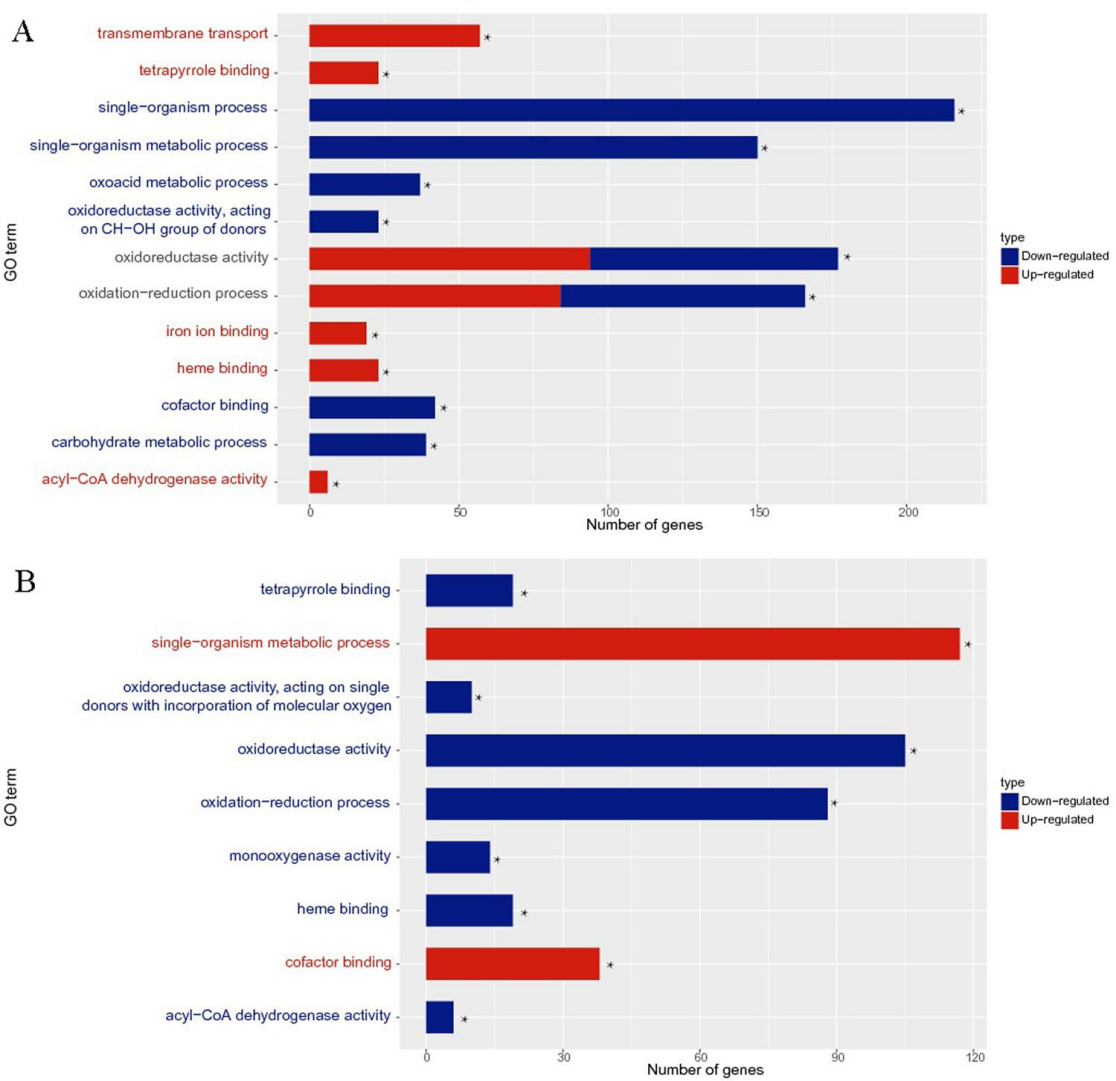
Enrichment of GO terms associated with differentially expressed genes. GO terms with corrected P-values < 0.05 were considered significantly enriched in terms of DEGs. The figure displays the most significantly enriched GO terms (★) linked to the DEGs found in the three groups of transcripts. (**A**) B15 vs D; (**B**) B60 vs B15. The horizontal axis represents the number of DEGs associated with the GO terms. Red: upregulated; blue: downregulated.

GO terms significantly enriched in DEGs that were upregulated in B15 vs D group included heme binding, tetrapyrrole binding, oxidoreductase activity, oxidation-reduction process, iron ion binding, acyl-CoA dehydrogenase activity, and transmembrane transport (Fig. 3(A); Supplementary Data S4). Meanwhile, GO terms enriched in DEGs that were downregulated in the B15 set included single-organism metabolic process, single-organism process, oxidation-reduction process, carbohydrate metabolic process, oxoacid metabolic process, cofactor binding, oxidoreductase activity, oxidoreductase activity, and acting on CH-OH group of donors (Fig. 3(A); Supplementary Data S5). These observations showed that significantly enrichment GO term in B15 vs D group mainly concentrated on oxidoreductase activity and oxidation-reduction process. Moreover, heme and tetrapyrrole are oxidoreductase cofactors and thus involved in redox reactions, supporting a theme in the GO results. Additionally, acyl-CoA dehydrogenase plays a key role in β-oxidation of fatty acids. It is clear that B15 light treatment induced expression of several genes involved in oxidation-reduction reactions in *M*. *prupureus* M9.

Comparing the B15 vs D and B60 vs B15 groups, we found that the significantly enriched GO terms downregulated by B15—cofactor binding and single-organism metabolic process—were upregulated by B60 (Fig. 3(B); Supplementary Data S6). Meanwhile, the major GO terms upregulated by B15 were downregulated by B60 (Fig. 3(B); Supplementary Data S7). These results indicated that B15 light treatment induced several oxidation-reduction reactions in M9, while the number of these reactions declined to levels typical of the D samples when exposure time is extended to B60.

**Figure 4:**
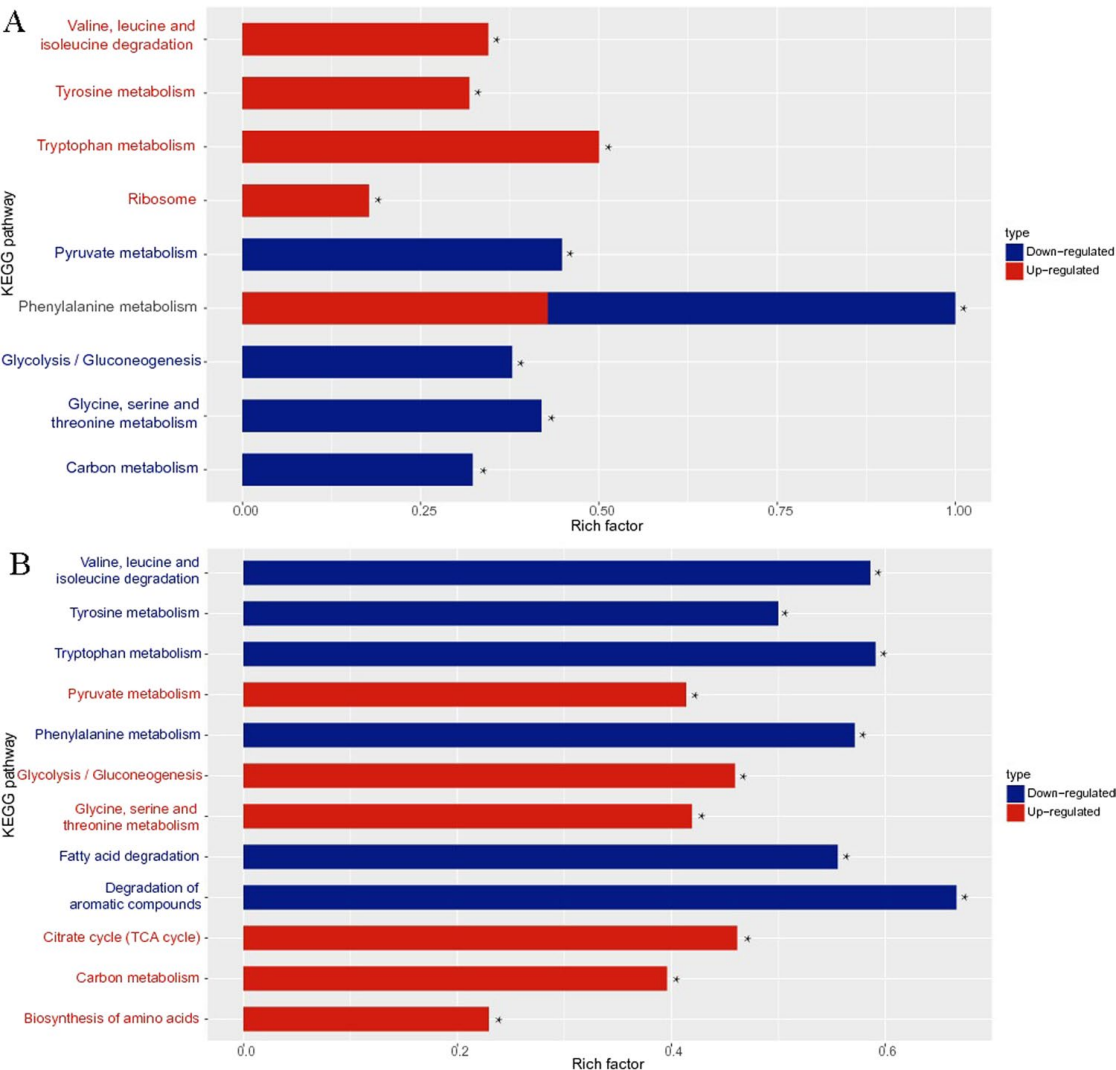
Enrichment of KEGG pathways involving differentially expressed genes. KEGG pathway analysis uses datasets to generate pathways illustrating metabolic and signal transduction mechanisms. Pathways with corrected P-values < 0.05 were considered significantly enriched in DEGs. The figure displays the KEGG pathways most significantly enriched (★) in DEGs corresponding to the transcripts in the three sample groups. (**A**) B15 vs D; (**B**) B60 vs B15. The enrichment factor represents the percent composition of transcripts representing DEGs versus background genes involved in the pathway. Red: upregulated; blue: downregulated.

KEGG pathway analysis revealed that DEGs were significantly enriched in metabolic and signal transduction pathways. Compared with D, B15 light treatment upregulated numerous DEGs, which were significantly enriched in pathways controlling tryptophan metabolism, valine, leucine and isoleucine degradation, ribosome, phenylalanine metabolism, and tyrosine metabolism (Fig. 4(A); Supplementary Data S8). Tryptophan, phenylalanine and tyrosine are aromatic amino acids and can be converted into several biological compounds by a series of oxidation reactions, including 5-hydroxy tryptamine, heteroauxin, dopamine, melanin involvement in physiology and growth of organism. Valine, leucine and isoleucine are branched chain amino acids; their degradation produces acetyl-CoA and methyl-CoA, which are synthetic precursors of type II polyketides such as *Monascus* pigments. Genes involved in the branched chain amino acid degradation pathway were upregulated in B15 samples, possibly indicating that *Monascus* pigment biosynthesis is induced by blue light.

The DEGs that showed significant enrichment within the KEGG pathway were upregulated in B15 vs D group; however, they were all downregulated in B60 vs B15 group (Fig. 4(B); Supplementary Data S11). Although genes involved in fatty acid and aromatic compound degradation were not considered to be upregulated in B15 vs D group—due to their corrected P-values being greater than or equal to 0.05—they were still downregulated in B60 vs B15 group (Fig. 4(B); Supplementary Data S11). Conversely, the significantly enrichment pathway which were downregulated in B15 vs D group (Fig. 4(A); Supplementary Data S9), were mostly upregulated in B60 vs B15 group pertained to carbon metabolism, glycolysis/gluconeogenesis, glycine, serine and threonine metabolism, and pyruvate metabolism (Fig. 4(B); Supplementary Data S10). Even though the P-values of genes involves in the citrate cycle (TCA cycle) and amino acid biosynthesis were not significantly downregulated in B15 vs D group, they were still upregulated in B60 vs B15 group (Fig. 4(B); Supplementary Data S10).

### Effects of blue light on primary metabolism of *Monascus purpureus* M9

According to KEGG pathway analysis, blue light exerted a global influence on the primary metabolism of *M. purpureus* M9. B15 light treatment reduced carbon and nitrogen metabolism while enhancing aromatic amino acid metabolism, associated with development of *Monascus*, and branched chain amino acid degradation and fatty acid degradation, with the last two taking part in polyketide biosynthesis. However, during B60 treatment, M9 upregulated carbohydrate metabolism and protein biosynthesis, apparently as part of its stress response to blue light.

### Analysis of differentially expressed genes involved in pigment biosynthesis

A majority of genes involved in *Monascus* pigment biosynthesis showed differential expression in the B15 vs D and B60 vs B15 comparisons, suggesting that pigment production is regulated by blue light. Expression of gene C5.129—a transcriptional regulator of the pigment biosynthesis gene cluster—as well as the genes comprising that cluster (C6.698, C1.1079, C2.21, C2.23, C2.29, and C2.31) was upregulated by blue light in the B15 set; with the exception of C5.138, those same genes were downregulated to their respective D levels when subjected to B60 treatment. This indicated that B15 light treatment was more favorable to inducing pigment biosynthesis than was B60 (Supplementary Table S1; Supplementary Data S12–17).

In our study, pigment yields were in accordance with gene expression. The color value of intracellular and extracellular *Monascus* pigments under B15 conditions were slightly higher than those under D conditions, while D and B60 color values were almost identical (Supplementary Fig. S3(A)). However, in our previous study, we observed significant differences in the concentrations of red, orange, and yellow pigments between the dark and the blue light-exposed samples^14^.

Genes encoding heme-thiolate proteins such as the cytochrome P450 protein family—which are associated with pigment biosynthesis—also undergo light-induced regulation. Expression of the genes encoding cytochrome P450 proteins were upregulated by B15 light treatment and downregulated to their D levels when subjected to B60 conditions. Similarly, the anabolic pathway of heme, a co-factor of polyketide metabolism, was also regulated by blue light in a manner similar to that seen with cytochrome P450.

### Analysis of differentially expressed genes involved in growth and reproduction

Of 27 DEGs related to development, eight genes linked to asexual sporulation were upregulated by B15 light treatment, while the rest were downregulated compared to their expression levels in the D sample (Supplementary Table S2; Supplementary Data S12–17). The downregulated genes included C7.619, which is involved in initiating mitosis^26^; C1.187, C1.813, and C3.795, which are associated with spindle formation^27–30^; and C1.635, C2.396, and C6.392, which are linked to the spindle formation checkpoint^31–33^. These responses suggest that exposure to B15 conditions slows development of M9 (Supplementary Table S2; Supplementary Data S12–17).

However, most of the aforementioned genes recovered to their D-state expression levels after exposure to B60 conditions. It was theorized that M9 slowed growth and reproduction activity as part of an effort to resist blue light-induced stress.

Differential gene expression related to sexual and asexual development in M9 was also observed in response to blue light stimulus. Some genes related to sexual development were downregulated in the B15 set when compared to the D sample, such as C3.689 (which is involved in the initiation of meiosis^34^), C1.277 (which also has a role in meiosis^35^), and C5.27, C7.467, C5.108, C4.214, C2.450, which take part in gametogenesis and spore wall assembly (Supplementary Table S2; Supplementary Data 12–17)^36, 37^. However, eight genes related to asexual development were upregulated in the B15 set. These included C6.584, which is involved in asexual growth^38^; C2.339, which contributes to conidium formation; C5.674 and C4.520, which are required for conidium cell wall organization; and C4.715, a gene essential for spore release and hyphal growth in response to stress (Supplementary Table S2; Supplementary Data 12–17). Gene expression measurements were accompanied by phenotypic observation: more conidia were formed under blue light versus the number generated in darkness. Conversely, few cleistothecia appeared when mycelia were exposed to blue light, strongly limiting the formation of ascopores (Supplementary Fig. S3(B)).

### Regulatory effect of blue light upon *Monascus* M9 IP3/Ca^2+^ and DAG/PKC signaling pathway

The gene C8.462—which was upregulated by B15 treatment— (0.61129 fold) encodes 1-phosphatidylinositol-4,5-bisphosphate phosphodiesterase 1(PLC1), an enzyme that degrades phosphatidylinositol-4,5- bisphosphate (PIP2), releasing the secondary messengers inositol-1,-4,5-trisphosphate (IP3) and diacylglycerol (DAG) (Supplementary Fig. S4; Supplementary Data S12). IP3 is involved in intracellular Ca^2+^ signaling in cells, a mechanism that regulates a host of physiological processes; DAG is a component in the protein kinase C (PKC) signal transduction pathway, which phosphorylates various proteins^39^. The most important signaling pathway in cells, the coupled IP3/Ca^2+^-DAG/PKC, was activated by B15 light treatment. Exposure to B15 conditions also upregulated the genes C3.606 (which encodes the calcium-transporting enzyme ATPase 2), C8.218 and C4.276 (which encode another calcium transport protein) and C1.416 (which codes for a calcium/calmodulin-dependent protein kinase); each of the aforementioned genes was downregulated to their D-state levels upon exposure to B60 treatment (Supplementary Data S12; Supplementary Data S17).

### MAPK signaling pathway is inhibited by blue light

Mitogen-activated protein kinase (MAPK) cascades are signal transduction pathways that are found throughout eukaryotes, including yeasts, animals and plants. MAP kinase pathways are composed of a MAP kinase, a MAP kinase kinase, and a MAP kinase kinase kinase; the system is regulated by sequential phosphorylation of its components. These protein phosphorylation cascades link extracellular stimuli to a wide range of cellular responses. Various physiological processes including conidial germination, hyphal fusion and carotenoid biosynthesis are controlled by MAPK cascades in *Neurospora crassa* ^40^, which also play a key role during development and secondary metabolism in *A. nidulans*, *A. niger* and *A. fumigatus* ^41, 42^. The genes C3.689 (−0.65148 fold), and C2.450— (−0.462 fold) both of which are involved in the MAPK pathway—were downregulated in the B15 sample, whereas C2.238 (0.7377 fold), C2.450 (0.8447 fold), and C4.409 (0.8098 fold) were upregulated in the B60 sample (compared to their expression levels in the B15 sample) (Supplementary Fig. S5; Supplementary Data S13– 15).

### Effects of blue light on transcriptional factors and global regulatory factors

Transcriptional factors (TFs) directly regulate gene expression and consequently play an important role in signal transduction. 18 differentially expressed TFs in *Monascus purpureus* M9 were activated by blue light; 15 belonged to the Zn2Cys6 protein family, two were of the C2H2 type and one was of the GATA type. The genes C5.761 and C3.1031 encode TFs that serve a regulatory function in nitrogen metabolism; C1.882 codes for a TF involved in carbon metabolism; and other TFs regulate various other metabolic pathways. Changes in TF gene expression were in accordance with the KEGG pathways, which indicates that blue light influences TFs expression (Supplementary Table S3; Supplementary Data 12–17).

### Validation of transcriptome data by RT-qPCR

Expression profiles obtained by RNA-seq analysis were validated by the selection of ten contigs (C1.416, C3.606, C4.276, C3.689, C2.450, C6.584, C5.674, C4.520, C1.187, C1.1079) involved in signaling pathways, reproduction, and pigment biosynthesis for RT-qPCR analysis, with β-actin serving as the reference gene. In every case, RT-qPCR data was consistent with the sequencing data (Supplementary Figure S6).

## Discussion

Light is an important signal for every living cell. In fungi, alterations in sexual development, conidiation, circadian clock resetting, hyperpolarization of the cell membrane, and other metabolic processes have been observed in response to light^9^. More recently, RNA-seq was used to identify components of light perception machinery and their downstream signaling cascades in fungi. *Neurospora* transcriptome sequencing revealed that light-induced genes can be approximately classified as early response (with peak mRNA levels between 15 and 45 min of light treatment) and late response (with peak mRNA levels between 45 and 90 min of light treatment)^23^. The GATA family transcription factor SUB-1 was required for the activation by light of late response genes^43^. The genes *frq* (the central clock component frequency) and *al-3* (mycelial carotenoid biosynthesis) were both early- and late-response genes^44^. However, the light-stimulated signal transduction pathway in *Monascus* was still unclear. To better understand the response of *Monascus* to blue light and its associated light-controlled gene regulatory network, we used transcriptome sequencing to identify *M. purpureus* M9 transcripts, then compared sequencing data from cells grown in darkness to cells exposed to blue light for 15 min and 60 min periods.

The results showed that blue light functions as a signal to modulate different physiological aspects in M9. Short-term exposure to blue light resulted in oxidative stress, which correlated with upregulation of genes encoding cytochrome C peroxidase (C8.314), peroxidase (C1.1127, C5.557, C8.171), and non-ribosomal peptide synthetase (C1.1079) (Supplementary Data S12). Conversely, there was a reduction in primary metabolic activity, including carbon and nitrogen metabolism; at the same time, M9 upregulated metabolic pathways involving aromatic amino acids, which is affiliated with development of M9, and branched chain amino acid degradation, which produces acetyl-CoA and malonyl-CoA, the biosynthetic precursors of pigments. Moreover, the β-oxidation of fatty acids was enhanced, providing ATP and NADH to fuel increased metabolic activity. It is notable that *Monascus* pigments play a role in protection against light damage in mycelia. In addition, it was observed that exposure to blue light led to increased conidia growth, possibly indicating that blue light induces a stress response in *Monascus* that promotes asexual reproduction (via spore formation) (Supplementary Fig. S3(B)). However, long-term exposure to blue light under B60 conditions induces *Monascus* to upregulate carbohydrate metabolism and protein biosynthesis, apparently as defensive measures against blue light-induced stress.

**Figure 5:**
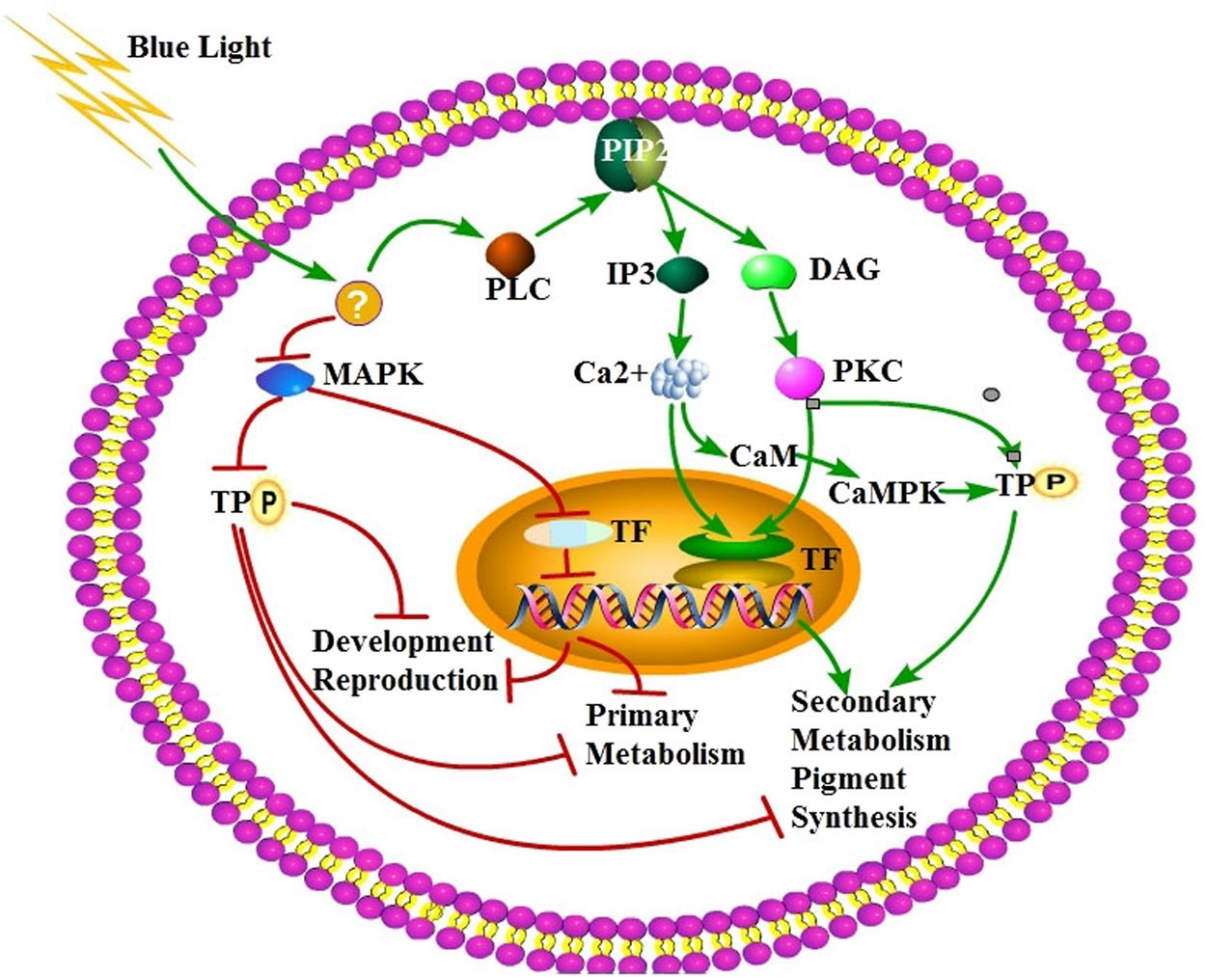
A putative blue light signaling pathway in *M. purpureus* M9. Red and blue arrows indicate inhibition and activation, respectively. Blue light activates the IP3-DAG signaling pathway in M9 while inhibiting the MAPK signaling pathway. Phosphatidylinositol 4, 5 bisphosphate (PIP2), located on cell membrane, was degraded by1-phosphatidylinositol 4, 5-bisphosphate phosphodiesterase (PLC), and liberated to inositol 1, 4, 5 trisphosphate (IP3) and diacylglycerol (DAG).The IP3-DAG pathway regulates the intracellular Ca^2+^ concentration; the MAPK signaling pathway is associated with development and secondary metabolism in many fungi. Genes encoding transcription factors (TFs) and target proteins (TP) are also regulated by the two signaling pathways above. Exposure to B15 conditions slowed primary metabolism, development and reproduction in M9 while enhancing secondary metabolism such as pigment biosynthesis.

Blue light exerted a widespread influence on *M. purpureus* M9. Based on our transcriptome analysis, we propose a possible blue light-induced signal transduction pathway in *Monascus* (Fig. 5). Short-term exposure to blue light (the B15 treatment) activated the IP3/Ca^2+^ and DAG/PKC signal transduction pathway and inhibited the MAPK pathway, while also regulating the biosynthesis of transcription factors and other proteins that impact metabolic processes. In response to B15 treatment, M9 reduced its primary metabolism and slowed development and reproduction, while upregulating secondary metabolism, especially biosynthesis of photoprotective pigments that absorb blue light. However, no photoreceptors, such as WC-1 and WC-2 in *N. crassa*, have been identified in *M. purpureus* M9.

Our study investigated the physiological responses of *Monascus* to blue light via transcriptome sequencing, revealing that the photoresponse of *M. purpureus* differs in notable ways from the well-studied system found in *N. crassa*. Therefore, this work should represent a general advancement in both photobiology and *M. purpureus* research. We propose a possible light-stimulated signal transduction pathway in *Monascus* and expect it to provide the foundation for future studies of the molecular mechanisms of *Monascus* light responses.

## Methods

### Cultivation of *Monascus* mycelium

*Monascus purpureus* strain M9, maintained on PDA slant at 30 °C, was reinoculated once a month. Spores suspension of M9 (adjusted to 10^6^ spores /ml) was harvested by sterile water and inoculated into rice medium (rice powder, 50 g/L; KH_2_PO_4_, 1.5 g/L; NaNO_3_, 3 g/L and MgSO_4_·7H_2_O, 1 g/L) in a 12-mm culture dish. The dishes were incubated at 30 °C without shaking in the darkness or exposed to blue light (100 lux) for 15 min/d, 60 min/d for 8 days, respectively. Light source is cool white illuminant of 465 nm without heating.

Fresh mycelia were obtained on the 8th day. Parts of mycelia were placed into 1.5 ml screw-cap tubes and snap-frozen in liquid nitrogen for RNA extraction. The remaining was used to morphology observation and pigments concentration analysis. Dry weight of mycelia was measured and pigment concentration was determined using a UV-visible spectrophotometer. Cleistothecia and conidia formations were observed by Olympus BH2 compound microscope.

### RNA extraction, library preparation and sequencing

Total RNA was extracted from frozen mycelia using the Plant RNA Kit (Omega, CA, USA). RNA degradation and contamination was monitored on 1% agarose gels. The purity was checked using the NanoPhotometer® spectrophotometer (IMPLEN, CA, USA). RNA concentration was measured using Qubit® RNA Assay Kit in Qubit® 2.0 Flurometer (Life Technologies, CA, USA) and integrity was assessed using the RNA Nano 6000 Assay Kit of the Bioanalyzer 2100 system (Agilent Technologies, CA, USA).

Total amount of 3 μg RNA per sample was used as input material for the RNA sample preparations. Sequencing libraries were generated using NEBNext® Ultra™ RNA Library Prep Kit for Illumina® (NEB, USA) following manufacturer’s recommendations and index codes were added to attribute sequences to each sample. Briefly, mRNA was purified from total RNA using poly-T oligo-attached magnetic beads. Fragmentation was carried out using divalent cations under elevated temperature in NEBNext First Strand Synthesis Reaction Buffer (5X). First strand cDNA was synthesized using random hexamer primer and M-MuLV Reverse Transcriptase (RNase H^−^). Second strand cDNA synthesis was subsequently performed using DNA Polymerase I and RNase H. Remaining overhangs were converted into blunt ends via exonuclease/polymerase activities. After adenylation of 3′ ends of DNA fragments, NEBNext Adaptor with hairpin loop structure were ligated to prepare for hybridization. In order to select cDNA fragments of preferentially 150–200 bp in length, the library fragments were purified with AMPure XP system (Beckman Coulter, Beverly, USA). Then 3 μl USER Enzyme (NEB, USA) was used with size-selected, adaptor-ligated cDNA at 37 °C for 15 min followed by 5 min at 95 °C before PCR. Then PCR was performed with Phusion High-Fidelity DNA polymerase, Universal PCR primers and Index (X) Primer. At last, PCR products were purified (AMPure XP system) and library quality was assessed on the Agilent Bioanalyzer 2100 system.

The clustering of the index-coded samples was performed on a cBot Cluster Generation System using TruSeq PE Cluster Kit v3-cBot-HS (Illumia) according to the manufacturer’s instructions. After cluster generation, the library preparations were sequenced on an Illumina Hiseq platform and 125 bp/150 bp paired-end reads were generated.

### Sequence reads assembly, mapping and annotation

Raw data of fastq format were firstly processed through in-house perl scripts. In this step, clean data were obtained by removing reads containing adapter, reads containing ploy-N and low quality reads from raw data. At the same time, Q20, Q30 and GC content the clean data were calculated. All the downstream analyses were based on the clean data with high quality.

The *Monascus purpureus* YY-1 reference genome and gene model annotation files were downloaded from genome website (http://spxy.tust.edu.cn/duxj/index.html) directly^25^. Index of the reference genome was built using Bowtie v2.2.3 and paired-end clean reads were aligned to the reference genome using TopHat v2.0.12.

Gene function was annotated based on the following databases: the NR (NCBI non-redundant protein sequences), Swiss-Prot (A manually annotated and reviewed protein sequence database), KEGG (KEGG Ortholog database), and GO (Gene Ontology), using BLAST with a cutoff E-value of 10^−5^.

### Quantification and differential expression analysis of transcripts

HTSeq v0.6.1 was used to count the reads numbers mapped to each gene. And then FPKM of each gene was calculated based on the length of the gene and reads count mapped to this gene.

Differential expression analysis of three conditions (two biological replicates per condition) was performed using the DESeq R package (1.18.0). DESeq provides statistical routines for determining differential expression in digital gene expression data using a model based on the negative binomial distribution^45^. The resulting P-values were adjusted using the Benjamini and Hochberg’s approach for controlling the false discovery rate. Genes with an adjusted P-value < 0.05 found by DESeq were assigned as differentially expressed.

### GO and KEGG enrichment analysis of differentially expressed genes

GO enrichment analysis of DEGs was implemented by the GOseq R package, in which gene length bias was corrected. GO terms with corrected P-value < 0.05 were considered significantly enriched by differential expressed genes^46^.

KEGG is a database resource for understanding high-level functions and utilities of the biological system from molecular-level information, especially large-scale molecular datasets generated by genome sequencing and other high-through put experimental technologies^47^. We used KOBAS software to test the statistical enrichment of differential expression genes in KEGG pathways^48^.

### Confirmation of gene expression level by RT-qPCR

First-strand cDNA was synthesized using the PrimeScript 1st Strand cDNA Synthesis Kit (TaKaRa, Japan), with the Oligo dT Primer 15. Gene expression was monitored by RT-qPCR and carried out using the SYBR Premix Ex Taq II (TaKaRa, Japan). RT-qPCR was performed using the Stratagen Mx3000 P (Agilent) with the following cycling program: hold at 95 °C for 30 s, followed by a three-step PCR (42 cycles of denaturation at 95 °C for 5 s, annealing at 60 °C for 30 s, and extension at 72 °C for 30 sec) and dissociation curve analysis (at 95 °C for 15 s, annealing at 60 °C for 30 s, then collecting the dissociation curve from 60 °C to 95 °C, finally at 95 °C for 15 s).

### Statistical analysis

For each sample, three technical replicates of the RT-qPCR assay were used with three biological replicates. Results were expressed as means ± standard deviation (SD) of the number of experiments. T-test for the values was performed at P < 0.05.

## Electronic supplementary material

Supplementary information

Dataset S1

Dataset S2

Dataset S3

Dataset S4

Dataset S5

Dataset S6

Dataset S7

Dataset S8

Dataset S9

Dataset S10

Dataset S11

Dataset S12

Dataset S13

Dataset S14

Dataset S15

Dataset S16

Dataset S17

Dataset S18
