# Supplementary material for: The molecular mechanisms of Monascus purpureus M9 responses to blue light based on the transcriptome analysis
Source: Sci Rep. 2017 Jul 17;7:5537. doi: 10.1038/s41598-017-05990-x (PMC5514072; doi:10.1038/s41598-017-05990-x)
Supplement: Supplementary file 1 — Supplementary information [file 41598_2017_5990_MOESM1_ESM.doc]

**The molecular mechanisms of *Monascus purpureus* M9 responses to blue light based on the transcriptome analysis**

**Di Chen^1,2^, Mianhua Chen^1^, Shufen Wu^1^, Zhenjing Li^1^, Hua Yang^1^, Changlu Wang^1*^**

^1^Key Laboratory of Food Nutrition and Safety（Tianjin University of Science and Technology）, Ministry of Education, College of Food Engineering and Biotechnology, Tianjin University of Science and Technology, No. 29, 13th Avenue, TEDA, Tianjin, 300457, P. R. China

^2^College of Biological Engineering, Henan University of Technology, No.100, Lianhua Street, High-tech Industrial Development Area, Zhengzhou, 450001, P. R. China

**Author Contributions:**

D. C. did the experiment. M. C. analyzed the data. D. C. prepared the figures and wrote the main manuscript text. C. W., S. W., Z. L. and H. Y. revised the text. All authors reviewed the manuscript.

***Corresponding author:**

Prof. Changlu Wang,  Key Laboratory of Food Nutrition and Safety（Tianjin University of Science and Technology）, Ministry of Education, College of Food Engineering and Biotechnology, Tianjin University of Science and Technology, No. 29, 13th Avenue, TEDA, Tianjin, 300457, China

E-mail: [clw123@tust.edu.cn](mailto:clw123@tust.edu.cn), Tel/Fax: ++86-22-60601154(O)

**Supplementary Tables**

**Supplementary Table S1. Differentially expressed genes related to pigments biosynthesis.**

| Gene | Function | log_2_FoldChange | | |
| --- | --- | --- | --- | --- |
|  |  | B15 vs D | B60 vs B15 | B60 vs D |
| C5.129 | Transcriptional regulatory protein | Down | Up | No change |
| C5.137 | Pigment biosynthesis polyketide synthase | Down | Up | No change |
| C6.861 | Lutein, carotenoid synthase | Up | Down | No change |
| C5.126 | Unknown | Up | Down | No change |
| C5.127 | Fatty acid synthase | Down | Up | No change |
| C5.138 | Unknown | No change | Up | Up |
| C6.698 | Polyketide- Nonribosomal peptide synthetase | Up | Down | No change |
| C1.1079 | Nonribosomal peptide synthetase | Up | Down | No change |
| C2.468 | L-aminoadipate-semialdehyde dehydrogenase large subunit | Down | Up | No change |
| C2.19 | Unknown | Down | Up | No change |
| C2.21 | Unknown | Up | Down | No change |
| C2.23 | Conidium pigment synthetic oxidase | Up | Down | No change |
| C2.26 | Unknown | Up | Down | No change |
| C2.29 | Unknown | Up | Down | No change |
| C2.31 | Unknown | Up | Down | No change |
| C2.32 | Unknown | Up | Down | No change |

**Supplementary Table S2. Differentially expressed genes related to reproduction and development.**

| Gene | Blast swiss prot | log_2_FoldChange | | | Involvement processes |
| --- | --- | --- | --- | --- | --- |
|  |  | B15 vs D | B60 vs B15 | B60 vs D |  |
| C5.27 | Sexual differentiation process protein isp4 | Down | - | - | Sexual differentiation process |
| C7.467 | Mating-type protein MAT-1 | Down | Up | - | Sexual differentiation process |
| C2.219 | Meiotic recombination protein SPO11 | Down | - | - | Asexual and Sexual |
| C5.108 | 1,3-beta-glucanosyltransferase gas2 | Down | Up | - | Involved in spore wall assembly |
| C4.214 | Serine protease EDA2 | Down | - | - | Gametgenesis |
| C1.187 | Serine/threonine-protein kinase ark1 | Down | Up | - | Spindle formation and chromosomal alignment |
| C1.635 | Checkpoint serine/threonine-protein kinase bub1 | Down | - | - | Spindle-assembly checkpoint and chromosomal alignment |
| C7.619 | Serine/threonine-protein kinase mph1 | Down | - | - | Initiating mitosis |
| C2.396 | G2/mitotic-specific cyclin-B | Down | Up | - | Cell cycle at the G2/M (mitosis) transition |
| C3.555 | metallothionein expression activator | Down | - | - | The transcription of genes required for cell separation |
| C6.392 | G2/mitotic-specific cyclin-4 | Down | - | - | Cell cycle at the G2/M (mitosis) transition |
| C1.277 | Meiotically up-regulated gene 70 protein | Down | Up | - | meiosis |
| C2.232 | Glucan endo-1,3-alpha-glucosidase agn1 | Down | Up | - | The degradation of the cell wall material |
| C1.813 | Kinesin-like protein KIF22 | Down | - | - | Spindle formation and the movements of chromosomes during mitosis and meiosis |
| C3.689 | Pheromone P-factor receptor | Down | - | - | Initiation of meiosis |
| C4.74 | Separin | Down | - | - | Chromosome segregation |
| C5.756 | G2-specific protein kinase nimA | Down | - | - | Spindle formation and the movements of chromosomes during mitosis and meiosis |
| C3.795 | WD repeat-containing protein slp1 | Down | Up | - | Spindle formation |
| C2.450 | Rho guanine nucleotide exchange factor scd1 | Down | Up | - | Mating and morphogenesis |
| C5.674 | - | Up | Down | - | Cell wall of asexual spore |
| C4.227 | glucan 1,3-beta-glucosidase A | Up | Down | Down | Spores formation and release |
| C4.520 | Chitin synthase D | Up | Down | - | Cell wall biosynthetic process |
| C5.719 | Mannan endo-1,6-alpha-mannosidase DCW1 | Up | Down | - | Cell wall biosynthetic process |
| C6.584 | Heterokaryon incompatibility protein 6, OR allele | Up | Down | - | Asexual reproduction |
| C4.715 | Protein SUR7 | Up | Down | Fasle | Spores formation |
| C3.409 | Meiotically up-regulated gene 14 protein | Up | Down | Fasle | Mitosis |
| C2.339 | conidiation-specific protein-8 | Up | Down | - | Asexual spores formation |

**Supplementary Table S3. Differentially expressed transcriptional factors.**

| Gene | Blast swiss prot | Type | log_2_FoldChange | | |
| --- | --- | --- | --- | --- | --- |
|  |  |  | B15 vs D | B60 vs B15 | B60 vs D |
| C7.646 | Uncharacterized transcriptional regulatory protein C530.11c | Zn2Cys6 | Up | Down | - |
| C4.831 | Uncharacterized transcriptional regulatory protein C1F7.11c | Zn2Cys6 | Up | Down | - |
| C3.618 | pH-response transcription factor pacC/RIM101 | C2H2 | Up | - | - |
| C4.858 | Cell wall integrity transcriptional regulator CAS5 | C2H2 | Up | Down | - |
| C7.171 | Transcriptional activator ARO80 | Zn2Cys6 | Up | Down | - |
| C3.1031 | Uncharacterized transcriptional regulatory protein C1F7.11c | Zn2Cys6 | Up | Down | - |
| C3.290 | Nitrogen assimilation transcription factor nirA | Zn2Cys6 | Up | Down | - |
| C1.116 | Cutinase transcription factor 1 beta | Zn2Cys6 | Up | Down | - |
| C4.182 | Uncharacterized transcriptional regulatory protein C1327.01c | Zn2Cys6 | Up | Down | - |
| C8.503 | Uncharacterized transcriptional regulatory protein C139.03 | Zn2Cys6 | Up | Down | - |
| C3.390 | Transcriptional regulatory protein moc3 | Zn2Cys6 | Up | Down | - |
| C3.814 | Putative transcriptional regulatory protein YJL206C | Zn2Cys6 | Up | Down | - |
| C5.129 | Uncharacterized transcriptional regulatory protein C1F7.11c | Zn2Cys6 | Down | - | - |
| C4.251 | Acetamidase regulatory protein | Zn2Cys6 | Down | - | - |
| C5.761 | Nitrogen regulatory protein areA | GATA | Down | - | - |
| C1.882 | Transcription activator of gluconeogenesis acuK | Zn2Cys6 | Down | Up | - |
| C1.358 | Regulator of drug sensitivity 2 | Zn2Cys6 | Down | Up | Up |
| C8.500 | Transcriptional activator protein acu-15 | Zn2Cys6 | Down | Up | - |

**Supplementary Figures**

**
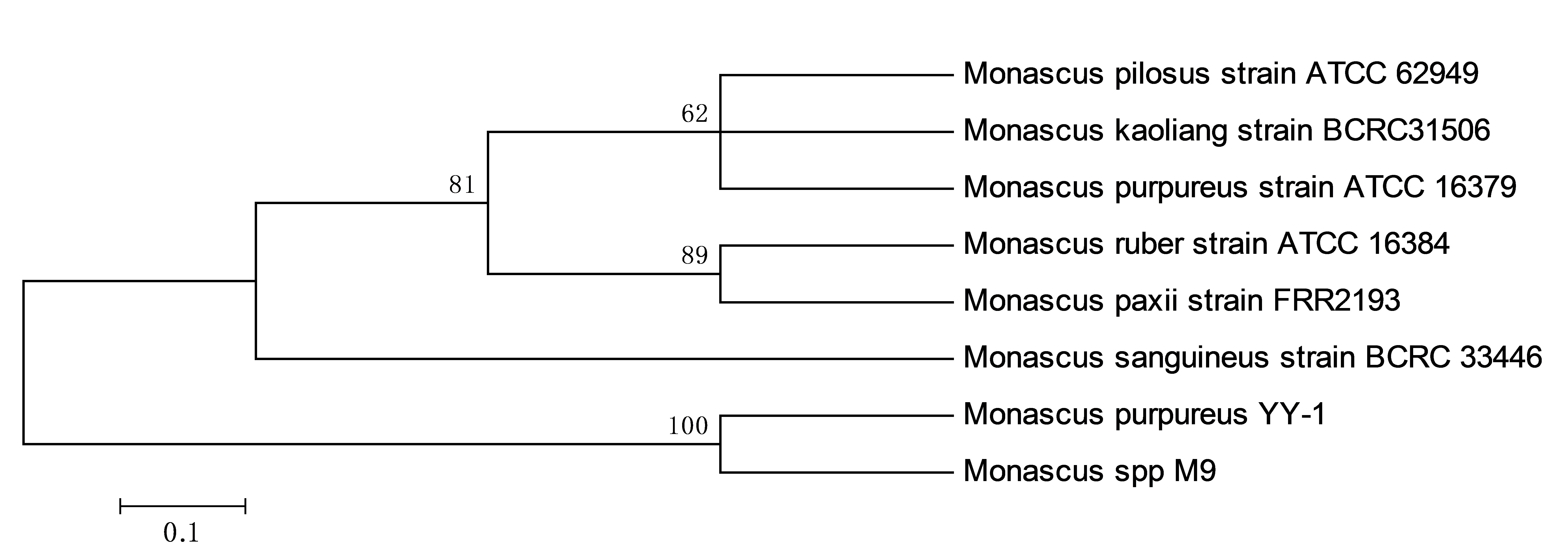
**

**Supplementary Figure S1.** **Phylogenetic analysis of the relationship between M9 and YY-1 in 8 *Monascus* spp.** ITS sequence of M9 was cloned and sequenced (Supplementary Data S18). 7 related *Monascus* spp. were selected to construct the phylogenetic tree using MEGA6 program with the maximum-likelihood method.


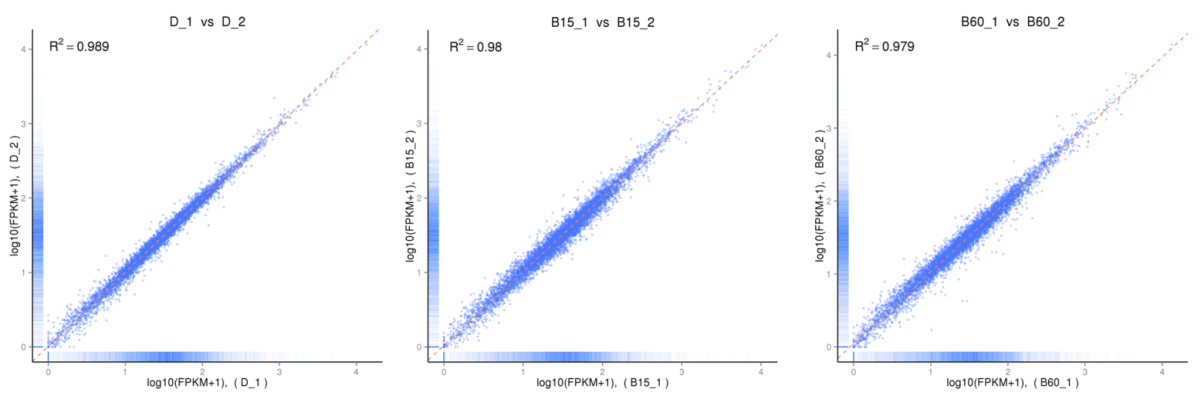


**Supplementary Figure S2. Comparison of transcriptome sequencing replicate experiments.** The FPKM for biological replicate 1 is plotted against biological replicate 2 for each gene, demonstrating strong correlation between replicate experiments at each time point. The correlation coefficient, R, is shown for each time point.


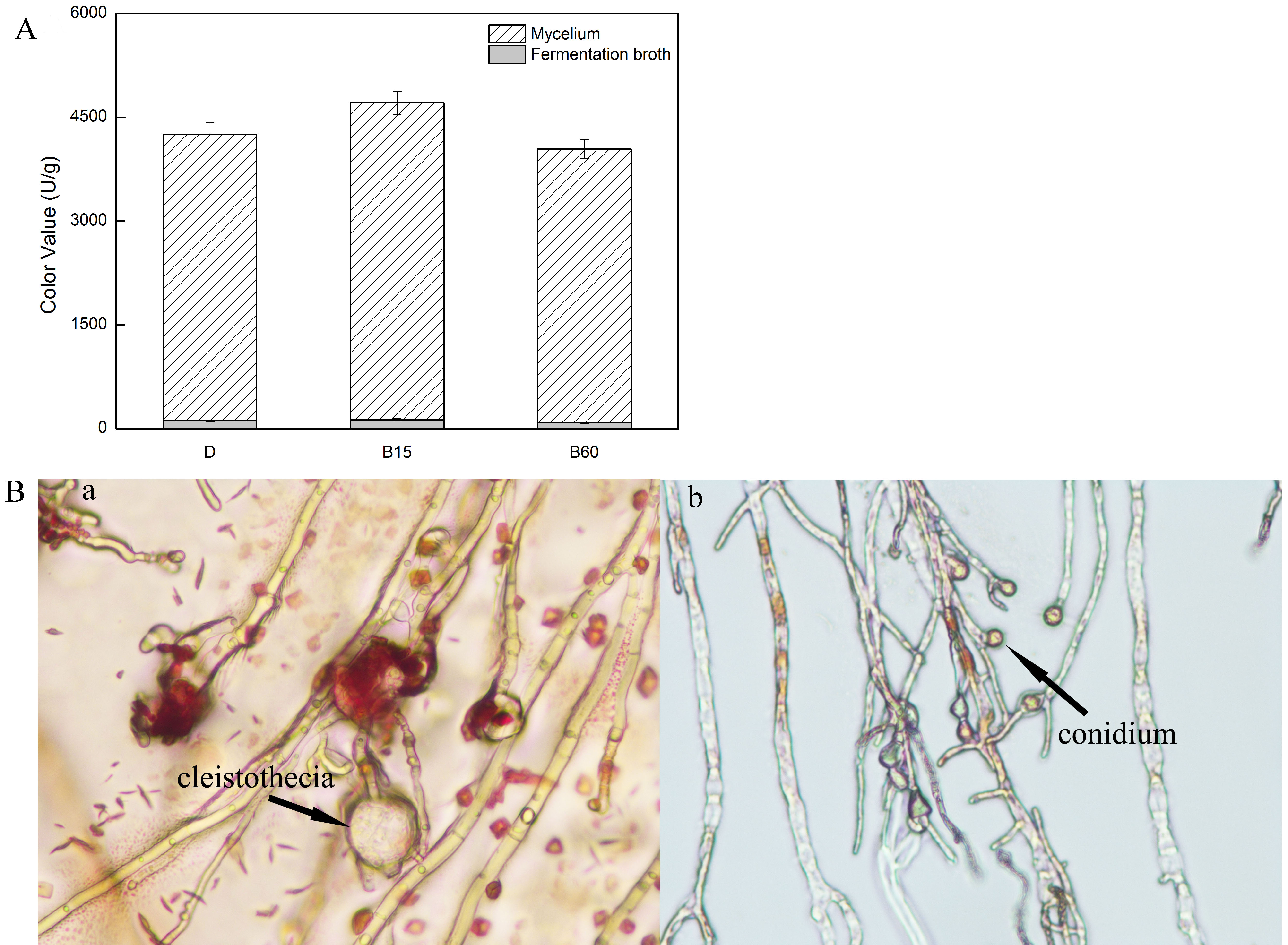


**Supplementary Figure S3. Pigments concentration and morphology observation of *M.purpureus* M9.** A: Pigments concentration of M9 under D, B15 and B60 condition. Color values of intracellular and extracellular extraction were determined by the absorbance at specific wavelength (505 nm). Pigments concentration was assessed by color values dividing by mycelium dry weight; B: Cleistothecia and conidia formation of M9 in the dark (a) or exposed to blue light (b).


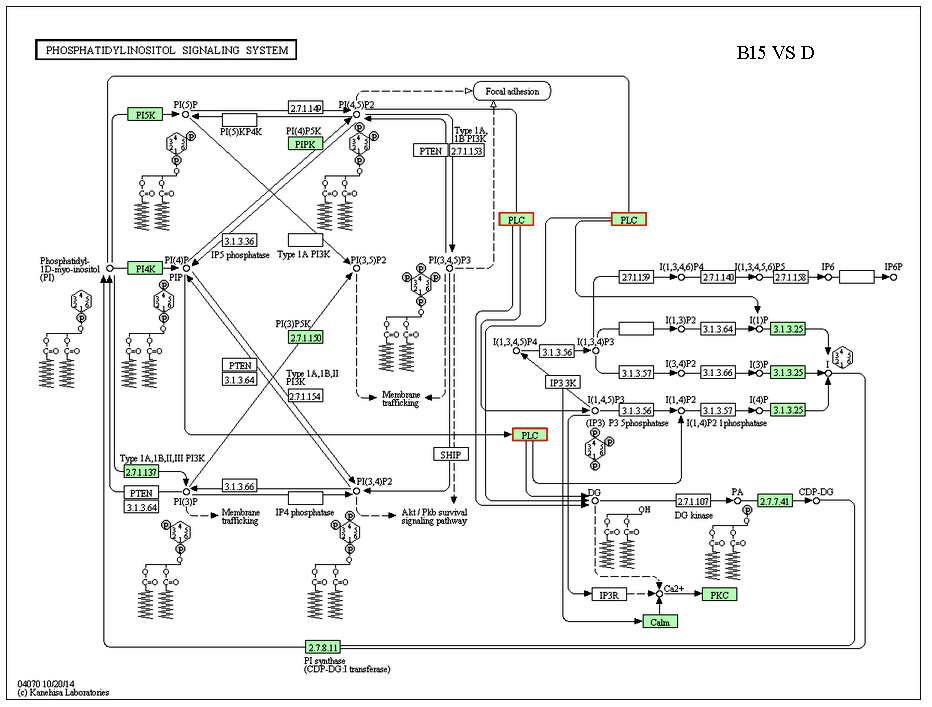


**Supplementary Figure S4.** **Phosphatidylinositol signaling system in sample B15 vs D.** Genes of green background were covered by reference genome; Red boxes indicate significantly increased expression. The image was obtained by KEGG.

**
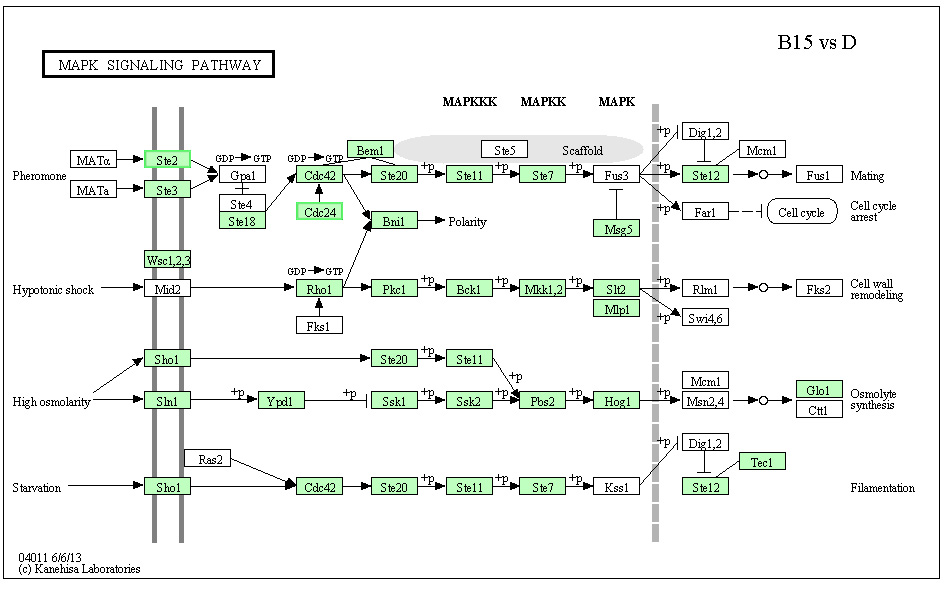
**

**
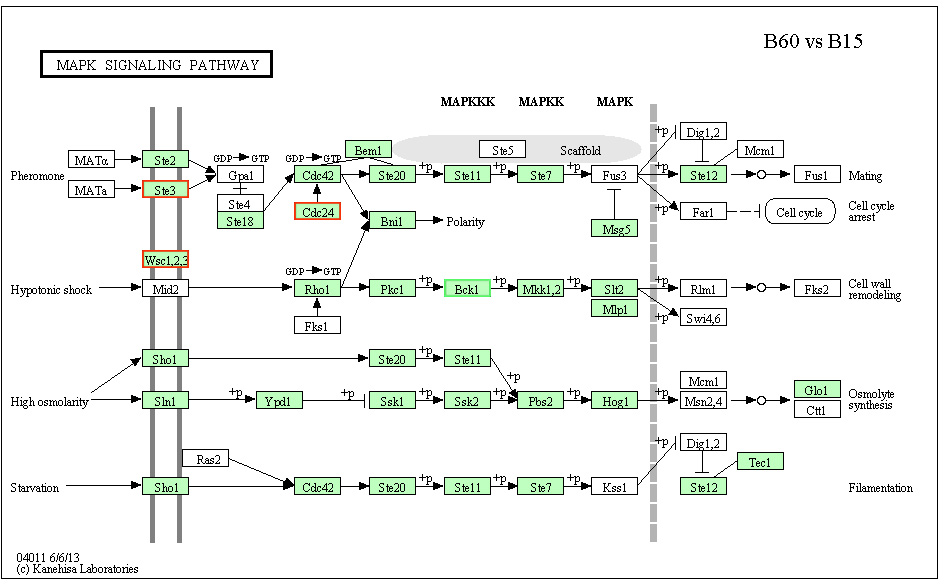
**

**Supplementary Figure S5.** **MAPK signaling pathway generated by KEGG analysis in samples B15 vs D and B60 vs B15.** Genes of green background were covered by reference genome; Red boxes and green boxes indicate significantly increased and decreased expression. These images were obtained by KEGG.

**
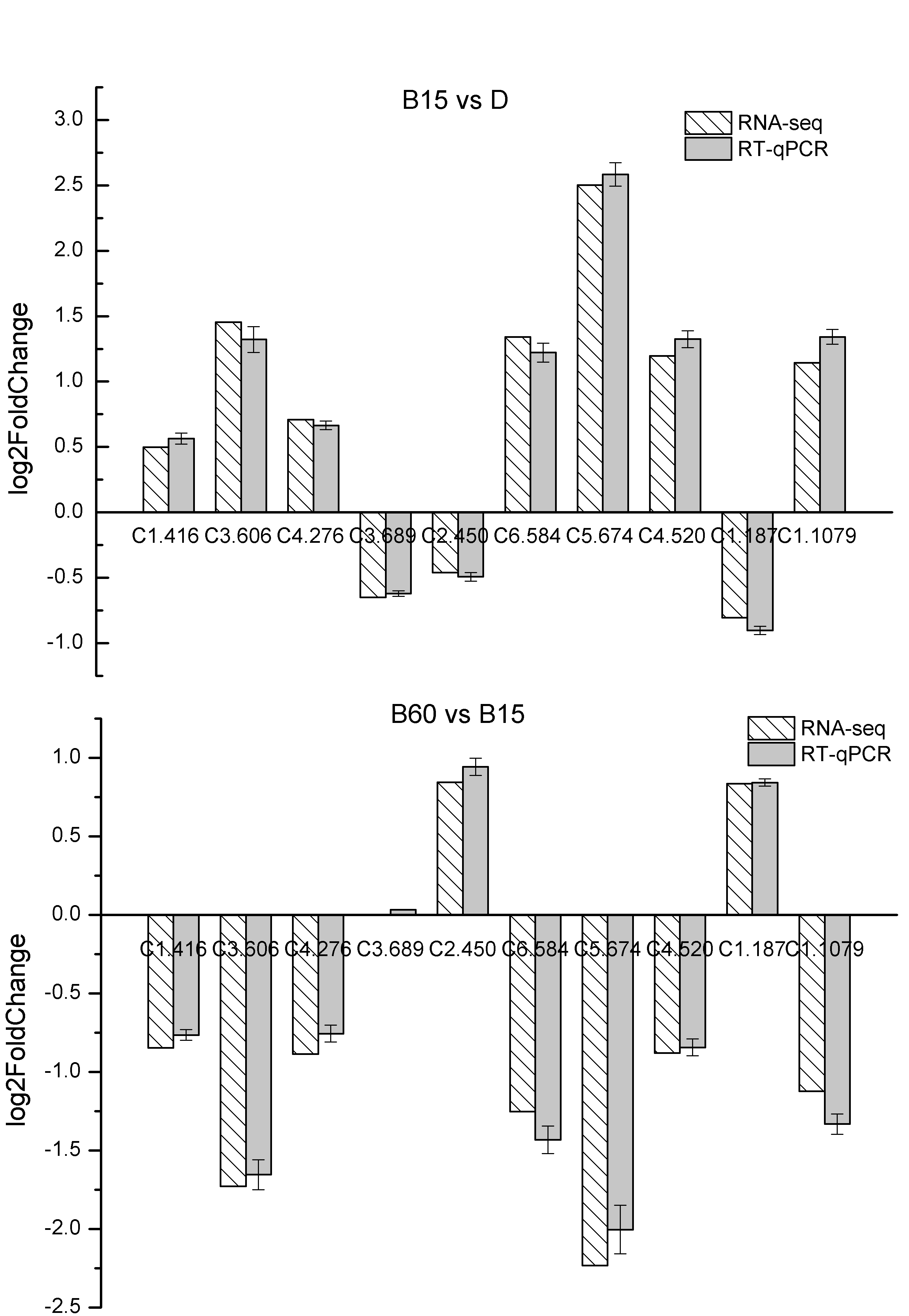
**

**Supplementary Figure S6. Comparison of the relative expression of the 10 genes by RT-qPCR and RNA-seq.**

**References**

1 Kanehisa, M., Furumichi, M., Tanabe, M., Sato, Y. & Morishima, K. KEGG: new perspectives on genomes, pathways, diseases and drugs. *Nucleic Acids Res.* **45**, D353-D361 (2017).

2 Kanehisa, M., Sato, Y., Kawashima, M., Furumichi, M. & Mao, T. KEGG as a reference resource for gene and protein annotation. *Nucleic Acids Res.* **44**, D457-D462 (2016).
