# Supplementary material for: The molecular mechanisms of Monascus purpureus M9 responses to blue light based on the transcriptome analysis
Source: Sci Rep. 2017 Jul 17;7:5537. doi: 10.1038/s41598-017-05990-x (PMC5514072; doi:10.1038/s41598-017-05990-x)
Supplement: Supplementary file 19 — Dataset S18 [file 41598_2017_5990_MOESM19_ESM.doc]

Primer:

ITS1 5’-TCCGTAGGTGAACCTGCGG-3’

ITS4 5’–TCCTCCGCTTATTGATATGC –3’

Sequencing result:

TTCCTCCGGCTTATTGATATGCTTAAGTTCAGCGGGTATCCCTACCTGATCCGAGGTCAACCTAAGGAAAAAAAGGTTGGAGAGGGCAAAGGCCCCGGCCCGACCTACTGAGCGGGTGACAAAGCCCCATACGCTCGAGGACCGGACGCGGCGCCGCCACTGCCTTTCGGGCCCGTCCCCGTTGCCCGGAGGCGCAGGGGACGGCGGCCCAACACACAAGCCGCGCTTGAGGGGCAGTAATGACGCTCGGACAGGCATGCCCCCCGGAATACCAGGGGGCGCAATGTGCGTTCAAAGATTCGATGATTCACTGAATTCTGCAATTCACATTACTTATCGCATTTCGCTGCGTTCTTCATCGATGCCGGAACCAAGAGATCCGTTGTTGAAAGTTTTAACCGATTTGGTATGTTTACTCAGACAGCAATCCTTTTCAAAGACAGCGTTCGAGAAGATGTCTCCGGCGGGCCCCAGGGGGCCGCGCCGAAGCAACAGGAGGTACAATAATCACGGGTGGGAGGTTGGGTCCCACGAAGGGGACCCGCACTCGGTAATGATCCTTCCGCAGGTTACCCTACGGAA
